# Dynamic Transcriptome Analysis Reveals Uncharacterized Complex Regulatory Pathway Underlying Genotype-Recalcitrant Somatic Embryogenesis Transdifferentiation in Cotton

**DOI:** 10.3390/genes11050519

**Published:** 2020-05-07

**Authors:** Huihui Guo, Haixia Guo, Li Zhang, Yijie Fan, Jianfei Wu, Zhengmin Tang, Yao Zhang, Yupeng Fan, Fanchang Zeng

**Affiliations:** State Key Laboratory of Crop Biology, College of Agronomy, Shandong Agricultural University, Tai’an 271018, China; hhguo@sdau.edu.cn (H.G.); diya_haixiaguo@163.com (H.G.); 15610418001@163.com (L.Z.); yjfan@sdau.edu.cn (Y.F.); jfwu@sdau.edu.cn (J.W.); 17861500710@163.com (Z.T.); yaozhang188@163.com (Y.Z.); fanyupeng@chnu.edu.cn (Y.F.)

**Keywords:** somatic embryogenesis, genotype-dependent response, embryogenic transdifferentiation, embryogenic competence, genotype-recalcitrant, dynamic regulatory pathway, molecular basis

## Abstract

As a notable illustration of totipotency and plant regeneration, somatic embryogenesis (SE) is the developmental reprogramming of somatic cells toward the embryogenesis pathway, the key step for genetic engineering. Investigations examining the totipotency process are of great fundamental and practical importance in crop biotechnology. However, high-frequency regeneration of cotton via SE has been limited due to genotype-dependent response. The molecular basis deciphering SE genotype recalcitrance remains largely unexplored in cotton. In the current study, to comprehensively investigate the dynamic transcriptional profiling and gene regulatory patterns involved in SE process, a genome-wide RNA sequencing analysis was performed in two cotton genotypes with distinct embryogenic abilities, the highly embryogenic genotype Yuzao 1 (YZ) and the recalcitrant genotype Lumian 1 (LM). Three typical developmental staged cultures of early SE—hypocotyls (HY), nonembryogenic calli (NEC) and primary embryogenic calli (PEC)—were selected to establish the transcriptional profiles. Our data revealed that a total of 62,562 transcripts were present amongst different developmental stages in the two genotypes. Of these, 18,394 and 26,514 differentially expressed genes (DEGs) were identified during callus dedifferentiation (NEC-VS-HY) and embryogenic transdifferentiation (PEC-VS-NEC), respectively in the recalcitrant genotype, 21,842 and 22,343 DEGs in the highly embryogenic genotype. Furthermore, DEGs were clustered into six expression patterns during cotton SE process in the two genotypes. Moreover, functional enrichment analysis revealed that DEGs were significantly enriched in fatty acid, tryptophan and pyruvate metabolism in the highly embryogenic genotype and in DNA conformation change otherwise in the recalcitrant genotype. In addition, critical SE-associated expressed transcription factors, as well as alternative splicing events, were notably and preferentially activated during embryogenic transdifferentiation in the highly embryogenic genotype compared with the recalcitrant genotype. Taken together, by systematically comparing two genotypes with distinct embryogenic abilities, the findings in our study revealed a comprehensive overview of the dynamic gene regulatory patterns and uncharacterized complex regulatory pathways during cotton SE genotype-dependent response. Our work provides insights into the molecular basis and important gene resources for understanding the underlying genotype recalcitrance during SE process and plant regeneration, thereby holding great promise for accelerating the application of biotechnology to cotton for improving its breeding efficiency.

## 1. Introduction

Somatic embryogenesis (SE) is a powerful tool for plant genetic improvement when used in combination with traditional agricultural techniques, and it is also an important technique to understand the different processes that occur during the development of plant embryogenesis [1]. Moreover, the regeneration of whole plants from differentiated cells cultured in vitro is a clear demonstration of the plasticity of plant cells [2]. In response to specific environmental signals, cells acquire competence to switch fate, which is accomplished by undergoing dedifferentiation and transdifferentiation processes followed by the implementation of a new developmental pathway [3]. The dedifferentiation process involves a terminally differentiated cell from its own lineage to a relatively low differentiated stage. This process allows cells to proliferate before redifferentiation. The process of transdifferentiation takes dedifferentiation a step further, in which cells can switch their lineages, allowing them to differentiate into another cell type [4,5].

Somatic embryogenesis plays an important role in cell fusion, genetic engineering, and somaclone variation. Many major crop varieties are known to be capable of somatic embryogenesis and the subsequent plant regeneration. Soybeans and cotton have proven to be the most difficult to regenerate [6,7]. To establish high-efficient embryogenic cultures that are stable and homogeneous, it is necessary to obtain information about the mechanisms involved in dedifferentiation and transdifferentiation, which are key developmental processes of the SE [8]. Moreover, the underlying mechanisms of somatic embryogenesis are significant for revealing important scientific theoretical problems such as cell development, differentiation, and morphogenesis [1,9,10,11]. Scientists have conducted extensive research on the mechanism of somatic embryogenesis, and the molecular events involved in the transition of a somatic cell to an embryogenic-competent cell have been investigated. A large number of genes are specifically expressed during somatic embryogenesis, and transdifferentiation is the result of the regulation of gene sequential expression [12,13,14,15,16,17,18,19,20].

SE is a complex process of molecular regulation in which somatic cells acquire totipotency to transform into embryogenic cells. Candidate specifically SE-related genes were identified. Results showed that *auxin response factor* (*ARF*) [21], *leafy cotyledon* (*LEC*) [22], *Wuschel* (*WUS*) [23], *somatic embryogenesis receptor kinase* (*SERK*) [24], and *baby boom* (*BBM*) [22] participated and played a decisive role in SE. In addition, the salicylic acid (SA) and jasmonic acid (JA) signaling pathways were also predicted to regulate SE [25]. During cotton SE, the high-mobility group box 3 (*GhHmgb3*) gene interference was reported to enhance embryogenic callus differentiation [26]. The specific AGP protein (GhPLA1) secreted from embryogenic callus was identified to enhance SE [27]. The casein kinase gene (*GhCKI*) negatively regulated SE process via a complex regulatory network [28]. Genes related to stress, such as *SERK1*, *abscisic aldehyde synthesis enzyme 2* (*ABA2*), *abscisic acid insensitive 3* (*ABI3*), *jasmonate ZIM-domain 1* (*JAZ1*), *late embryogenesis abundant protein 1* (*LEA1*), and transcription factors were also involved in callus induction [29]. Transcriptome sequencing was carried out during callus dedifferentiation and transdifferentiation. Auxin and stress response molecules were found to be involved and played an important regulatory role in cotton SE [30,31].

Although the ability to regenerate whole plants from cells, tissues, or organs cultured in vitro has long been known, the question of how a somatic cell can differentiate into a whole plant has been considered to be one of the most important questions facing science over the next quarter-century [32,33]. SE is difficult to obtain in some species due to genetic variabilities [3,34,35]. Moreover, in some crops, the SE efficiency has been compromised by the genotype recalcitrance, which in some cases, is a major problem to be resolved. How different plant genotypes can result in a variety of phenotypic outcomes under the same in vitro culture conditions has not been thoroughly elucidated to date.

Upland cotton is one of the most important economic crops, and the earliest commercialized transgenic crop worldwide [19]. Genetic engineering and tissue culture approaches have been widely applied in cotton biotechnology breeding. Cotton transgenic technology relies heavily on somatic embryogenesis [13,36]. However, genotypes have a decisive influence on somatic embryogenesis in cotton. The genotype of the material is the first factor to affect the somatic embryogenesis and plant regeneration of cotton, followed by the type of phytohormone and its concentration [36,37]. More than 100 cotton varieties have been used for tissue culture research. Furthermore, approximately half of these materials have embryogenic ability, while the remaining half are unable or extremely unlikely to induce somatic embryos [36,38,39]. Most cultivars have been recalcitrant, and few reports of high-frequency regeneration in cotton via SE have come forth, due to a genotype-dependent response [36,40,41,42]. Two cotton varieties with different SE capabilities were analyzed by high throughout RNA sequencing at three callus dedifferentiation induction stages during SE [16]. Transcriptomic analysis revealed that protein kinase and oxidoreductase activity were highly enriched during the same and different treatment stages between two cotton varieties. The *SERK* gene showed different expression patterns between the two varieties. Moreover, several stress-related transcription factors and the complex auxin and ethylene signaling pathway might contribute to differentiation in SE [16]. However, the dynamic transcriptional regulation characteristics during embryogenic transdifferentiation, which is the key SE developmental process, have not been investigated.

Cotton SE is the concerted process involving multiple cellular pathways controlled by a complicated gene regulatory network. High-frequency regeneration of cotton via SE has been limited due to the genotype-dependent response. The regulatory networks and molecular basis underlying SE genotype recalcitrance remains largely unexplored in cotton. In this study, a genome-wide transcriptome sequencing (RNA-seq) was performed to comprehensively investigate the dynamic transcriptomic profiles and gene regulatory patterns involved in the process of cotton somatic embryogenesis in two genotypes with distinct embryogenic abilities. By comprehensively analyzing the transcriptomic data, we were able to identify a set of differentially expressed genes at molecular levels, and to draw picture of events underpinning the SE development. Our work provides an important molecular basis and gene resources for elucidating the SE genotype recalcitrance during cell totipotency and further expanding crop genetic engineering.

## 2. Materials and Methods

### 2.1. Plant Materials and Culture Conditions

Upland cotton (*Gossypium hirsutum* cultivars cv. LM-1 and *Gossypium hirsutum* cultivars cv. YZ-1) seeds were surface sterilized with 0.1% HgCl_2_ (w/v) for 8 min and rinsed 3-4 times with distilled water. Seeds were then germinated on Murashige and Skoog (MS) medium supplemented with 3% (w/v) sucrose and 0.25% (w/v) phytagel. Hypocotyls (HYs) were cultured on MS plus B_5_ vitamin (MSB) medium containing 0.45 μmol·L^−1^ 2,4-dichlorophenoxyacetic acid (2,4-D) and 0.46 μmol·L^−1^ kinetin (KT). Nonembryogenic calli (NEC) were maintained on MSB medium without any hormone to induce primary embryogenic calli (PEC), as described by Guo et al. [43]. The embryogenic nature of cotton cell strains was determined by our previous research [44]. HYs (dedifferentiation induction 0 day), NEC (dedifferentiation induction ~45 days) and PEC (transdifferentiation initiation) samples were collected and frozen immediately in liquid nitrogen with three biological replicates for the following transcriptomic profiling. 

### 2.2. Library Construction for RNA Sequencing

The RNA extraction method was operated according to the instructions of the Aidlab^®^ Plant RNA Extraction Kit (RN38), from HY, NEC and PEC samples in the two genotypes. RNA purity was first checked using the kaiaoK5500^®^ Spectrophotometer (Kaiao, Beijing, China). RNA integrity and concentration were assessed using the RNA Nano 6000 Assay Kit of the Bioanalyzer 2100 system (Agilent Technologies, CA, USA). A total amount of 2 μg RNA per sample was used as input material for the RNA sample preparations. Sequencing libraries were generated using the NEBNext^®^ Ultra^TM^ RNA Library Prep Kit for Illumina^®^ (E7530L, NEB, USA) following the manufacturer’s recommendations, and index codes were added to attribute sequences to each sample. Briefly, mRNA was purified from total RNA using poly-T oligo-attached magnetic beads. Fragmentation was carried out using divalent cations under elevated temperature in NEBNext First Strand Synthesis Reaction Buffer. First strand cDNA was synthesized using random hexamer primer and RNase H. Second strand cDNA synthesis was subsequently performed using buffer, dNTPs, DNA polymerase I and RNase H. The library fragments were purified with QIAquick PCR kits and eluted with EB buffer, and then terminal repair, A-tailing and adapter added were implemented. The whole library was completed by PCR amplification and then sequenced on an Illumina platform (provided by Annoroad Gene Technology Co., Ltd., Beijing, China) with 150 bp paired-end reads. 

### 2.3. RNA-Seq Data and Gene Regulatory Pattern Analysis

Due to the limited amount of available materials of embryogenic transdifferentiation and unqualified sequencing distribution uniformity for a few replicates were observed among the samples. Considering unified comparison analysis in parallel for all samples in this study and repeatability of the RNA sequencing results, two ideal consistent replicates from each sample were used for subsequent transcriptome analysis. The reference genome file was downloaded from the CottonGen database (https://www.cottongen.org/species/Gossypium_hirsutum/nbi-AD1_genome_v1.1). Bowtie2 v2.2.3 was used for building the genome index, and clean transcriptome data were then aligned to the reference genome using TopHat v2.0.12. The reads count for each gene in each sample was counted by HTSeq v0.6.0, and RPKM (reads per kilobase million mapped reads) was then calculated to estimate the expression level of genes in each sample. DESeq2 v1.6.3 was used to estimate the expression level of each gene in per sample by linear regression and calculate the p-value with the Wald test. Finally, the p-value was corrected by Benjamini and Hochberg’s method. Genes with q ≤ 0.05 and |log2_ratio| ≥ 1 were identified as differentially expressed genes (DEGs). The normalized sequencing data from two biological replicates were used for DEGs identification. The GO (Gene Ontology, http://geneontology.org/) enrichment of DEGs was implemented by the hypergeometric test, in which p-value was calculated and adjusted as *q*-value. GO terms with *q* < 0.05 were considered to be significantly enriched. The KEGG (Kyoto Encyclopedia of Genes and Genomes, http://www.kegg.jp/) enrichment of DEGs was implemented by the hypergeometric test, in which *q*-value was adjusted by multiple comparisons as q-value. KEGG terms with *q* < 0.05 were considered to be significantly enriched. PlantTFDB (Plant Transcription Factor Database) was used for transcription factor identification. ASprofile v1.0.4 classified alternative splicing into 12 types and was used to present different AS events for each sample.

## 3. Results

### 3.1. Landscape of RNA Transcriptome During Cotton SE in Two Genotypes

To comprehensively analyze the gene regulatory patterns involved in SE process, we performed a genome-wide analysis of transcripts using the high throughput RNA-seq technology in two cotton genotypes with distinct embryogenic abilities, Yuzao 1 (YZ) with a high embryogenic ability (embryogenic transdifferentiation rate >80%,) and Lumian 1 (LM) with a very low ability (embryogenic transdifferentiation rate <10%). Three typical developmental staged cultures of early SE—hypocotyls (HY), nonembryogenic calli (NEC) and primary embryogenic calli (PEC)—were selected to establish the transcriptional profiles.

**Figure 1:**
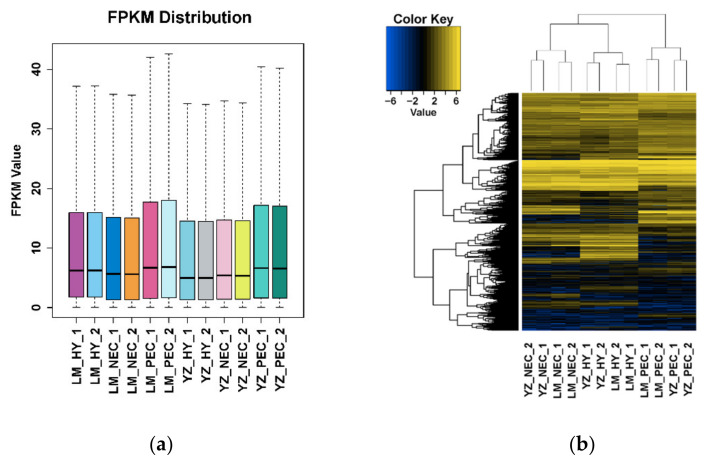
Overall data analysis for the transcriptome assays during cotton SE in two genotypes. (**a**) Fragments Per Kilobase Million (FPKM) distribution in different developmental stages during Somatic embryogenesis (SE) in Lumian 1 (LM) and Yuzao 1 (YZ); (**b**) Hierarchical clustering of transcripts in different developmental stages during early SE in LM and YZ.

The output results showed that more than 6 Gb RNA-seq clean data were filtered individually, with a high mapping rate (>94%) (Table S1). The quantitative expression levels for total 62,562 transcripts were calculated as FPKM (fragments per kilobase per million mapped fragments) based on the number of uniquely mapped reads that overlapped with exon regions [45] (Table S2). The FPKM distribution stability provided an important guarantee of the reliability of our transcriptome data (Figure 1a). Meanwhile, transcripts in the two genotypes were combined to perform a hierarchical clustering. We found that callus dedifferentiation and embryogenic transdifferentiation periods were clustered separately (Figure 1b), indicating that there were different gene expression patterns during the two developmental processes.

### 3.2. Differentially Expressed Gene Regulatory Patterns During Cotton SE

**Figure 2:**
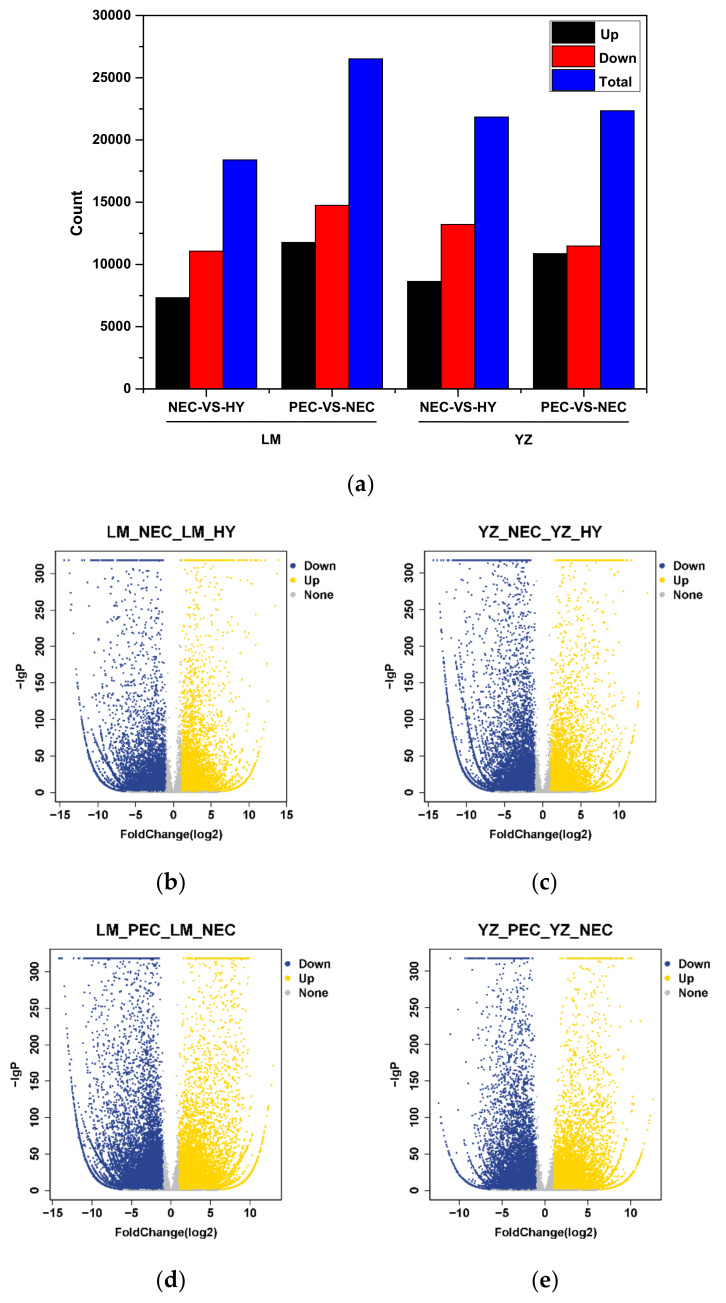

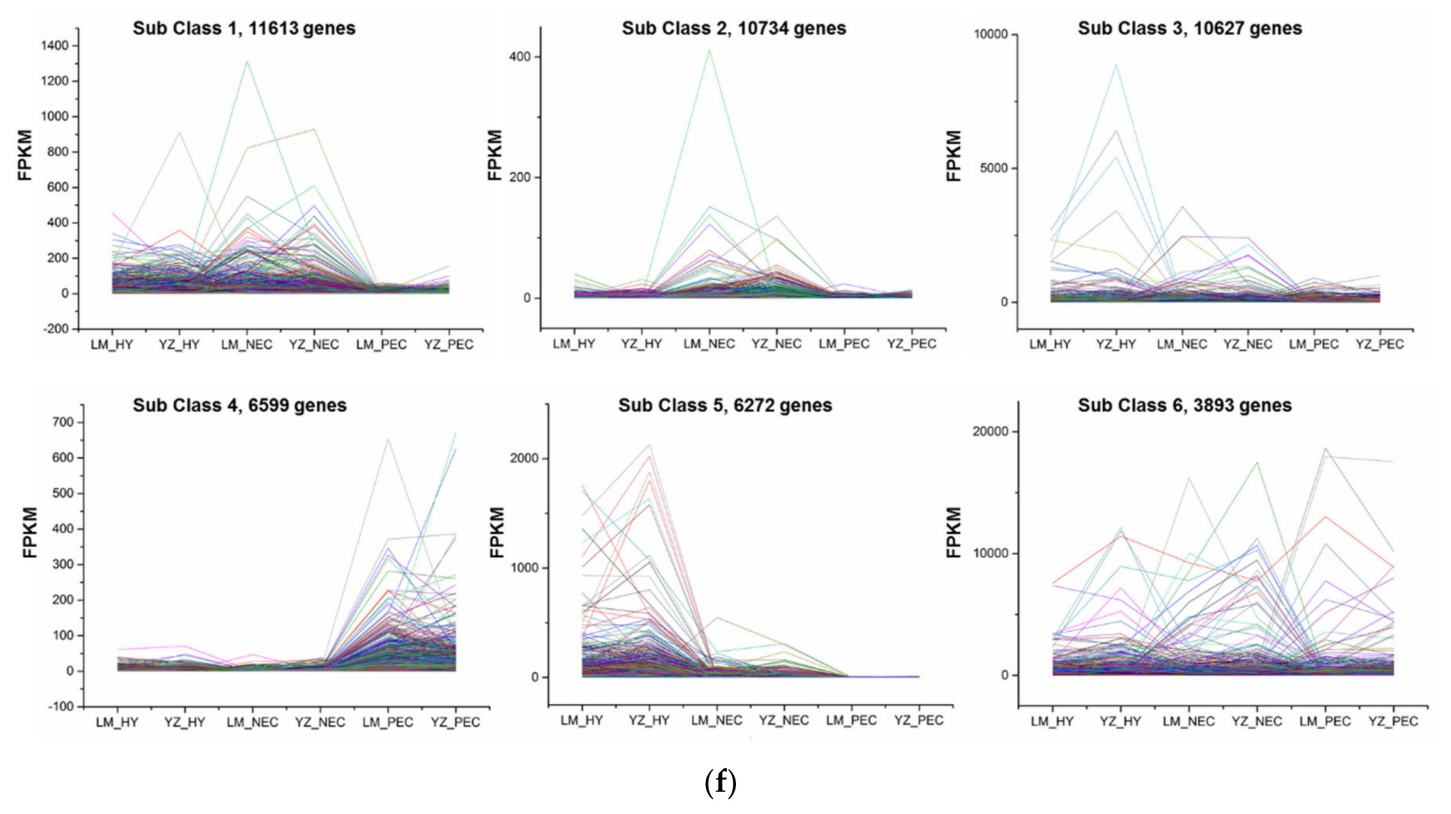
Analysis of differentially expressed genes in different developmental stages during cotton SE. (**a**) Identified differentially expressed genes (DEGs) during callus dedifferentiation and embryogenic callus transdifferentiation periods; (**b**) Volcano map of DEGs during callus dedifferentiation in LM; (**c**) Volcano map of DEGs during callus dedifferentiation in YZ; (**d**) Volcano map of DEGs during embryogenic callus transdifferentiation in LM; (**e**) Volcano map of DEGs during embryogenic callus transdifferentiation in YZ; (**f**) K-means clustering of DEGs in different developmental stages during early SE in LM and YZ. Colored lines indicate changes in gene expression levels.

The false discovery rate (FDR) was used to determine the threshold of p-values in multiple tests, which corresponded to the differential gene expression test [46,47]. In this study, q < 0.05 and the absolute value of |Log2Ratio| ≥ 1 was used as a threshold to judge the significant differences in gene expression. A total of 18,394, 26,514, 21,842, and 22,343 differentially expressed genes (DEGs) were identified in LM_NEC-VS-LM_HY, LM_PEC-VS-LM_NEC, YZ_NEC-VS-YZ_HY and YZ_PEC-VS-YZ_NEC, respectively (Figure 2a; Table S3). The results showed that compared to LM, more DEGs were expressed during callus dedifferentiation (Figure 2b,c), whereas fewer DEGs were expressed during the embryogenic transdifferentiation process in YZ (Figure 2d,e).

In addition, by k-means clustering, the differential genes were divided into several similar expression patterns. Results suggested that DEGs were clustered into six groups during the cotton SE process in two genotypes (Figure 2f; Table S4). Of these groups, the genes in class 4 were more highly expressed in the PEC stage compared to HY and NEC and positively correlated with the embryogenic transdifferentiation process. In contrast, genes were severely repressed in PEC stage in class 5 and were active to callus dedifferentiation during early SE.

### 3.3. Functional Enrichment Analysis of DEGs During Embryogenic Transdifferentiation in Two Genotypes

#### 3.3.1. DNA Conformation Change Involved in the Recalcitrant Genotype

**Figure 3:**
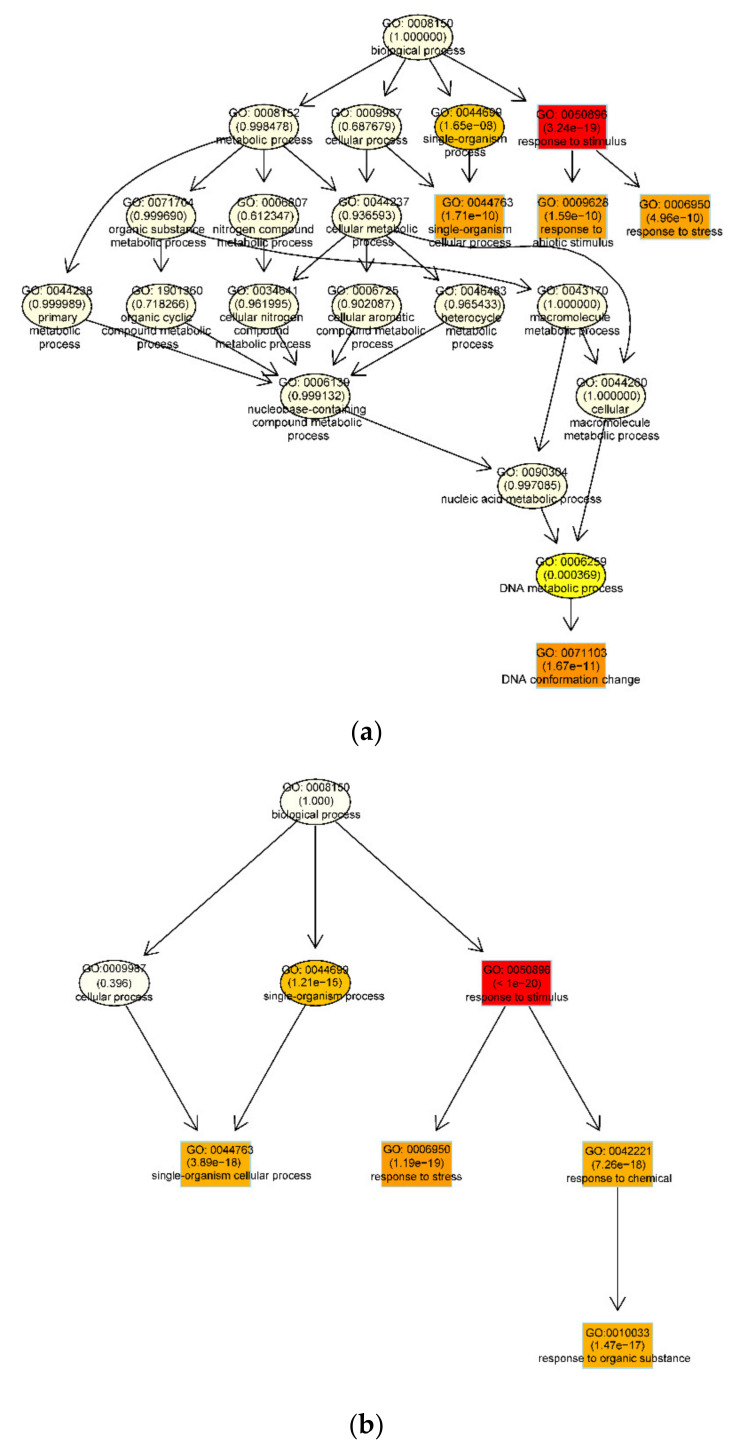
Functional GO enrichment analysis of differentially expressed genes (DEGs) during embryogenic transdifferentiation in the two cotton genotypes. (**a**) GO enrichment of DEGs in LM_PEC-VS-LM_NEC; (**b**) GO enrichment of DEGs in YZ_PEC-VS-YZ_NEC. The biological processes with a false-discovery rate adjusted *p*-value < 0.05 are shown. GO, Gene Ontology. Each node represents a GO term, and the boxes represent the top five GO terms. The color depth represents the enrichment degree. The redder the color, the higher the enrichment degree, the more yellow the color, the lower the enrichment degree. The name of the GO term and the adjusted *p*-value of enrichment analysis are shown on each node.

To investigate the functioning of differentially expressed genes, Gene Ontology (GO) analysis investigated DEG enrichment of biological processes during embryogenic transdifferentiation in the two genotypes [48] (Figure 3; Table S5). Strong enrichment in different biological processes were observed (*p* < 0.05). In LM, the recalcitrant genotype, DEGs were highly clustered in ‘response to stimulus’, ‘single -organism cellular process’, ‘response to abiotic stimulus’, ‘response to stress’ and ‘DNA conformation change’ processes (Figure 3a). In YZ, the highly embryogenic genotype, ‘response to stimulus’, ‘single-organism cellular process’, ‘response to stress’, ‘response to chemical’ and ‘response to organic substance’ processes were significantly enriched (Figure 3b). Regarding the most significant difference in the two genotypes, a high cluster pointed to ‘DNA conformation change’ was significantly observed in the recalcitrant genotype. The results were consistent with the report that transcription variations were more modulated by DNA methylation in the recalcitrant genotype [49].

We identified the DEGs enriched in DNA conformation change process during embryogenic differentiation in the two genotypes (Table 1). Of these, several major factors that are associated with DNA-binding, DNA helicase, sister chromatid cohesion, nucleosome assembly, structural maintenance of chromosomes, transcription initiation, cold shock, signal activation, cell division, histone, DNA gyrase, structural maintenance of chromosomes, DNA annealing helicase and endonuclease, DNA replication licensing factor, DNA replication ATP-dependent helicase, increased DNA methylation, cis-trans isomerase and E3 ubiquitin-protein ligase, were significantly observed. Of these, *GYRB* (DNA gyrase subunit B, chloroplastic/mitochondrial), *ZRANB3* (DNA annealing helicase and endonuclease), *MCM3* (DNA replication licensing factor), *HMGIY2* (HMG-Y-related protein A), and *FKBP53* (Peptidyl-prolyl cis-trans isomerase) were specifically activated. *IGHMBP2* (DNA-binding protein SMUBP-2), *SRS2* (ATP-dependent DNA helicase SRS2-like protein), *SYN1* (sister chromatid cohesion 1 protein 1), *NAP1* (nucleosome assembly protein 1), *TFG2* (transcription initiation factor IIF subunit beta), *CSP1* (cold shock protein 1), *PHRF1* (PHD and RING finger domain-containing protein 1), *ASCC3* (activating signal cointegrator 1 complex subunit 3), and *CDC45* (cell division control protein 45 homolog) were specifically downregulated during embryogenic differentiation process in LM, the recalcitrant genotype (Table 1).

#### 3.3.2. Fatty Acid, Tryptophan and Pyruvate Metabolism Involved in the Highly Embryogenic Genotype

**Figure A1:**
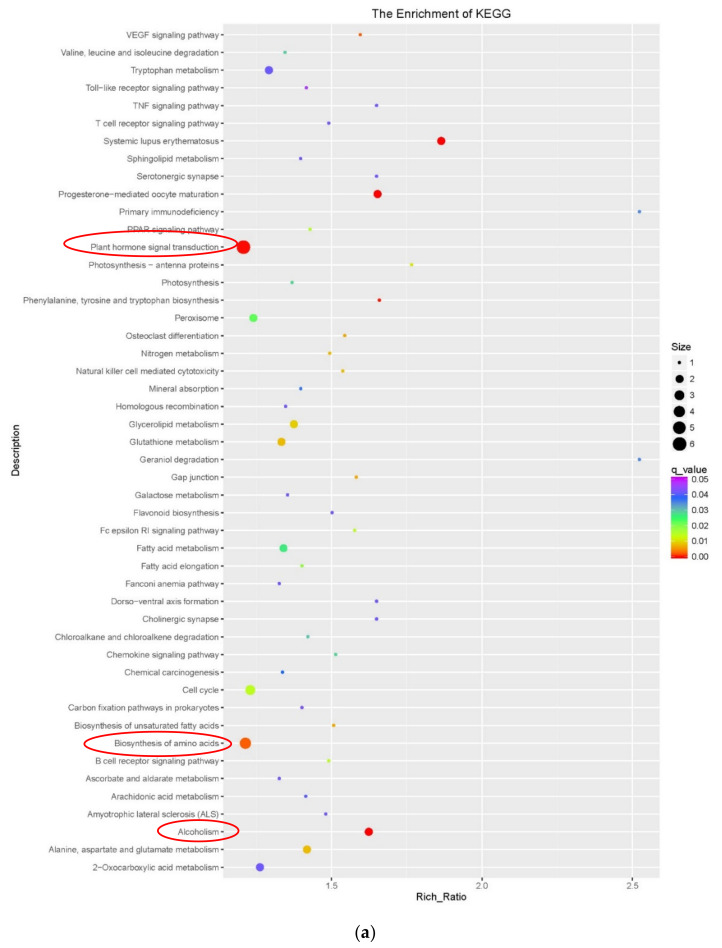

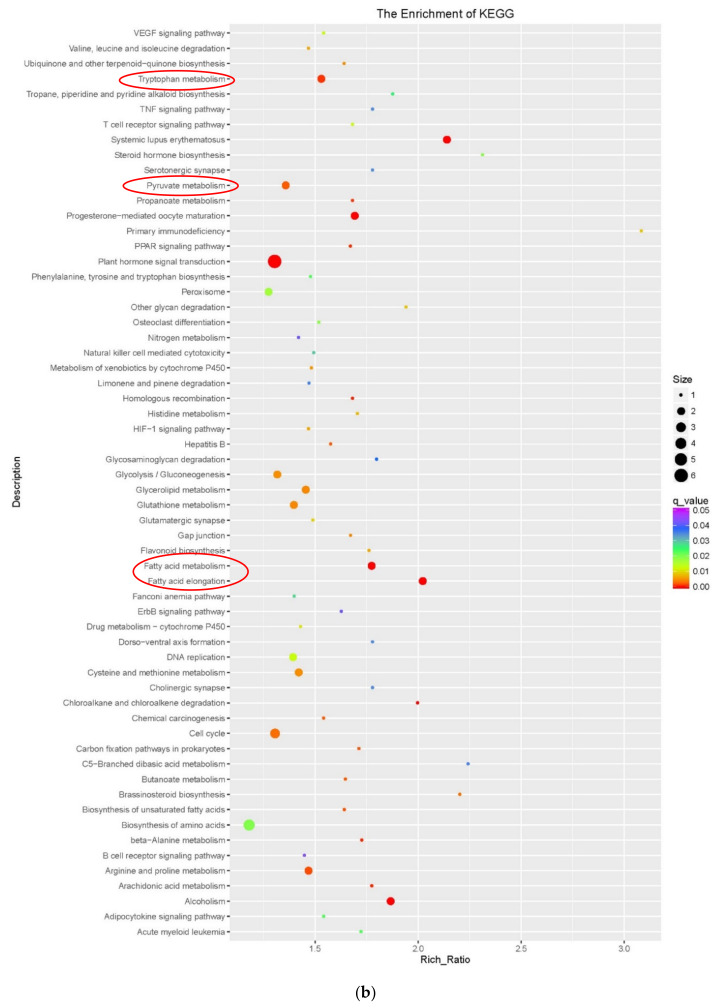
Functional KEGG enrichment analysis of differentially expressed genes during embryogenic transdifferentiation in the two genotypes. (**a**) KEGG enrichment of DEGs in LM_PEC compared to LM_NEC; (**b**) KEGG enrichment of DEGs in YZ_PEC compared to YZ_NEC. The KEGG pathway with a false-discovery rate adjusted p-value < 0.05 are shown. KEGG, Kyoto Encyclopedia of Genes and Genomes.

To further understand the function of SE-related genes, we analyzed the differences and dynamic changes of gene clustering by KEGG (Kyoto Encyclopedia of Genes and Genomes) enrichment analysis [50] (Figure A1; Table S6). Results showed that the terms ‘plant hormone signal transduction’, ‘biosynthesis of amino acids’, and ‘alcoholism’ pathways were significantly enriched in LM, the recalcitrant genotype (Figure A1a). While in YZ, the highly embryogenic genotype, DEGs were highly accumulated in ‘plant hormone signal transduction’, ‘tryptophan metabolism’, ‘pyruvate metabolism’, ‘fatty acid metabolism’, ‘fatty acid elongation’, ‘arginine and proline metabolism’ and ‘alcoholism’ pathways (Figure A1b). It is worth noting that fatty acid metabolism were significantly observed in the highly embryogenic genotype. The results in the highly embryogenic genotype were consistent with the report that a lipid transfer protein (SELTP) drives the totipotency of somatic cells in cotton [43].

The representative DEGs significantly enriched in fatty acid metabolism and fatty acid elongation processes during embryogenic differentiation in the two genotypes were listed in Table 2. Several major factors that are associated with enoyl-CoA hydratase, palmitoyl-protein thioesterase, 3-ketoacyl-CoA synthase, very-long-chain 3-oxoacyl-CoA reductase, very-long-chain (3R)-3-hydroxyacyl-CoA dehydratase, very-long-chain enoyl-CoA reductase, long chain acyl-CoA synthetase, acyl-coenzyme A oxidase, aldehyde dehydrogenase and alcohol dehydrogenase were significantly observed. Of these, *PPT3* (palmitoyl-protein thioesterase 3), *ECR* (very-long-chain enoyl-CoA reductase) were specifically activated, and *ADH6* (alcohol dehydrogenase 6) was specifically downregulated during embryogenic differentiation process in YZ, the highly embryogenic genotype (Table 2). 

We also identified the DEGs significantly involved in tryptophan metabolism and pyruvate metabolism processes during embryogenic differentiation in the two genotypes (Table 2). The representative factors, including L-tryptophan-pyruvate aminotransferase, indole-3-pyruvate monooxygenase, indole-3-acetaldehyde oxidase, cytochrome P450, UDP-glycosyltransferase, aldehyde dehydrogenase, phosphoenolpyruvate carboxylase, phosphoenolpyruvate carboxykinase (ATP), pyruvate kinase, dihydrolipoamide dehydrogenase, dihydrolipoamide acetyltransferase, acetyl-CoA synthetase and acetyl-CoA acetyltransferase were investigated. Of these, *UGT* (UDP-glycosyltransferase), *DLAT* (dihydrolipoamide acetyltransferase) were specifically activated, and *CYP* (Cytochrome P450), *AAT1* (Acetyl-CoA acetyltransferase) were specifically downregulated during embryogenic differentiation process in YZ, the highly embryogenic genotype (Table 2). 

### 3.4. Stress-Related Transcription Factors Were Specifically Activated and Preferentially Expressed During Embryogenic Transdifferentiation in the Highly Embryogenic Genotype

**Figure 4:**
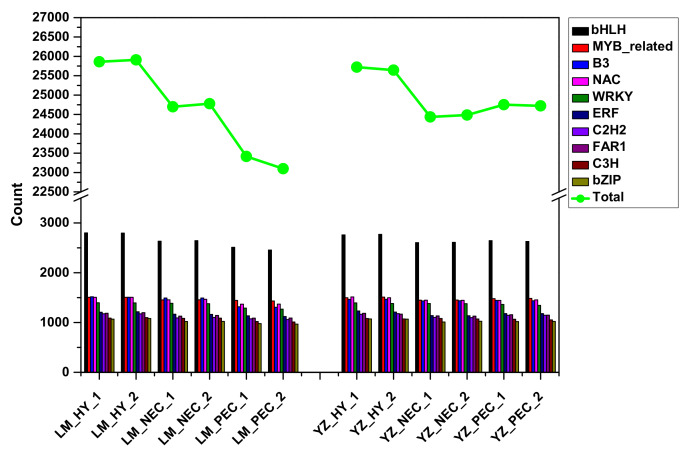
Transcription factors involved in different developmental stages during cotton SE in the two genotypes.

Transcription factors (TFs) are a group of protein molecules that can specifically bind to a specific sequence of a gene, which can ensure that the target gene is expressed in a specific time and space. TFs during early SE were explored in the two genotypes. The results showed that bHLH, followed by MYB-related, B3, NAC and WRKY, was identified and highly expressed among the samples (Figure 4; Table S7). Moreover, during the embryogenic transdifferentiation process, the number of TFs in YZ was higher than that in LM.

Previous studies confirmed that these transcription factors were differentially expressed under stress conditions and acted as a link between stress and the developmental pathway [51]. bHLH participates in the process of adaptation to various stresses. MYB-related is involved in hormone and environmental responses. B3 plays an important role in responsing to various stresses. NAC is involved in defensive responses to biotic and abiotic stresses. WRKY participates in responsing to biotic and abiotic stresses and hormone signaling.

In the process of cotton somatic embryogenesis, a large number of stress-related transcription factors were specifically activated and preferentially expressed during embryogenic transdifferentiation in the highly embryogenic genotype. These critical SE-associated transcription factors might play a significant role in regulating the stress response during embryogenic transdifferentiation.

### 3.5. Active Alternative Splicing Significantly Involved in Embryogenic Transdifferentiation in the Highly Embryogenic Genotype

**Figure 5:**
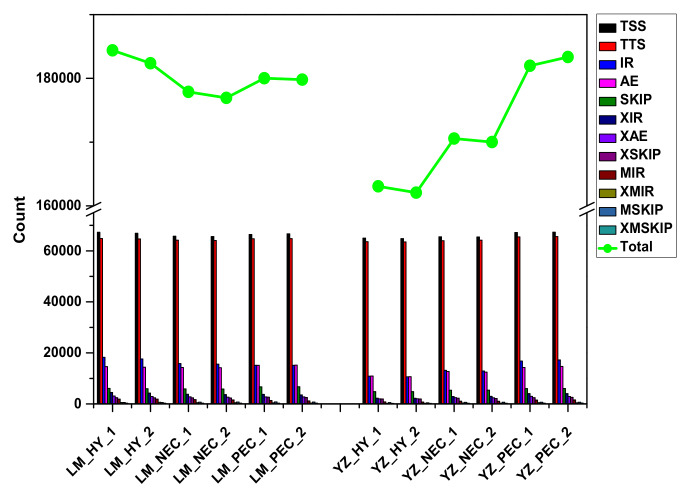
Alternative splicing events in different developmental stages during cotton SE in the two genotypes. TSS, transcript start site. TTS, transcript end site. IR, intron retention. AE, alternative exon end. SKIP, skipped exon. XIR, IR-like. XAE, AE-like. XSKIP, SKIP-like. MIR, Multi-IR. XMIR, MIR-like. MSKIP, Multi-exon SKIP. XMSKIP, MSKIP-like.

Various RNA splicing isomers can be produced by different splicing modes or different splicing sites. This process is alternative splicing (AS). AS exists widely in eukaryotes and is an important mechanism for regulating gene expression and protein diversity [52]. There are five types of AS events: alternative exon end (AE), intron retention (IR), skipped exon (SKIP), transcript start site (TSS) and transcript end site (TTS). The AS events during early SE were classified and quantitatively counted in the two genotypes (Figure 5). The results showed that TSS and TTS accounted for the highest proportion followed by IR and AE. During the embryogenic transdifferentiation process, compared with LM, the AS events continued to rise in YZ. Results suggested that the active AS events might play a positive role during embryogenic transdifferentiation in the highly embryogenic genotype.

## 4. Discussion

Plant in vitro propagation, such as somatic embryogenesis, has been possible because plant cells have the capacity to regenerate a whole organism from differentiated somatic cells [9,16,53]. Moreover, SE is an important tool for clonal propagation of important economical and agronomical species [54]. The high-frequency regeneration of cotton via SE has been limited due to genotype-dependent response. The molecular basis underlying SE genotype recalcitrance remains largely unexplored in cotton. During dedifferentiation induction of cotton SE, Cao et al. performed a high throughout RNA sequencing to analyze gene expression patterns in two cotton varieties with different SE capabilities [16]. Transcriptomic analysis revealed that protein kinase and oxidoreductase activities were highly enriched between two the cotton varieties. Several stress-related transcription factors and the auxin and ethylene signaling pathways might contribute to differentiation in SE. Moreover, the *SERK* gene showed different expression patterns between the two varieties [16]. However, the dynamic transcriptional regulation characteristics during embryogenic transdifferentiation have not been investigated so far. Compared with this report, in our current study, two cotton genotypes with distinct embryogenic abilities (YZ and LM), at three typical developmental staged cultures of early SE—hypocotyls, nonembryogenic calli and primary embryogenic calli—were selected to establish the transcriptional profiles. We found that DEGs identified in callus dedifferentiation and embryogenic transdifferentiation were clustered into six expression patterns during cotton SE process in the two genotypes. Moreover, functional enrichment analysis revealed that DEGs were significantly enriched in fatty acid, tryptophan and pyruvate metabolism in the highly embryogenic genotype and in DNA conformation change otherwise in the recalcitrant genotype. In addition, critical SE-associated expressed transcription factors, as well as alternative splicing events, were notably and preferentially activated during embryogenic transdifferentiation in the highly embryogenic genotype compared with the recalcitrant genotype.

The realization of plant high-frequency regeneration via SE depends largely on whether somatic cells can re-differentiate into embryogenic state. Our present work focused on the dynamic transcriptional regulation characteristics during embryogenic transdifferentiation, which is the key SE developmental process, with the expectation to provide a better understanding of the SE genotype recalcitrance during cell totipotency at molecular levels. 

### 4.1. Effects of DNA Conformation Change During Embryogenic Transdifferentiation

In our current study, to investigate the function of differentially expressed genes, we performed GO enrichment of biological processes during embryogenic transdifferentiation in two genotypes (Figure 3; Table S5). Results suggested that a strong cluster related to ‘DNA conformation change’ was significantly observed in LM, the recalcitrant genotype (Figure 3a). 

The effects of DNA conformation change in SE process have been demonstrated previously. In Scots pine (*Pinus sylvestris* L.) SE cultures, the cellular polyamine contents, which may provide protection by inducing conformational changes in DNA were analyzed throughout SE induction. Results revealed that manipulation of stress response pathways, such as changes in polyamine metabolism may provide a way to enhance somatic embryo production in recalcitrant lines [55,56]. In addition, molecular differences between cell lines previously characterized in terms of their embryogenic potential as embryogenic (E) and non-embryogenic (NE) were investigated during *Pinus radiata* SE process. Furthermore, the detection of vibrational markers of DNA conformation indicated that DNA samples obtained from E and NE lines presented different conformation, with the common B-DNA conformation in E samples, while Z-conformation in NE samples [57].

Moreover, Corredoira et al. reported that among the molecular mechanisms involved in regulation of SE, DNA methylation is an epigenetic modification of the chromatin that leads to a transcriptionally inactive conformation and gene silencing [58]. Cell reprogramming, totipotency and somatic embryogenesis initiation involve changes in the developmental genetic program of the cell, which affects global genome organization; in this sense, epigenetic modifications constitute key factors of genome flexibility and may be involved in these genome organization changes [58,59]. In this study, the *IDM1* (Increased DNA methylation 1) gene, which was enriched in DNA conformation change process, was significantly upregulated during embryogenic transdifferentiation in LM, the recalcitrant genotype (Table 1). The findings were consistent with our previous report that global DNA hypomethylation was observed during embryogenic redifferentiation in YZ [49]. To differentiate nonembryogenic and embryogenic cells, the mechanism of DNA methylation could be utilized as a candidate marker. DNA hypomethylation might play a positive role in regulating cotton somatic embryogenesis. In *Pinus nigra*, low methylation levels were observed in embryogenic cell lines [60,61]. The *Arabidopsis* SE was also encouraged by global DNA hypomethylation during the induction process [23]. The role of DNA methylation and the embryogenic capacity in *Chestnut* (*Castanea sativa* M.) have been evaluated. It was found that embryogenic competence could be established under in vitro conditions with DNA demethylation [62]. The loss in DNA methylation level was observed during *Coffea canephora* embryogenesis. The decrease in DNA methylation levels was related to the onset of dedifferentiation and cellular proliferation [63]. The low level of DNA methylation promotes gene expression associated with cell dedifferentiation. The reduction in the DNA methylation level permitted the triggering of cell dedifferentiation by expressing related genes. Because the lowest level of DNA methylation was always found in the embryogenic cells, it was possible that DNA hypomethylation was involved in the signals that lead to the induction of SE. The decrease in DNA methylation levels could be an important step during vegetative-embryogenic transition, in which somatic cells acquired the competency to form embryonic cells. The above results reveal the significant role of DNA conformation change in embryogenic cultures proliferation. 

### 4.2. Fatty Acid Metabolism Involved in Embryogenic Transdifferentiation

As protective chemicals in reproduction and seed preservation, fatty acids are the major structural components of membrane phospholipids and triacylglycerol storage oils, which play a significant role in the evolution and developmental processes in plants [15]. In the current study, the differences and dynamic changes of gene clustering by KEGG enrichment were analyzed to understand the function of SE-related genes in two cotton genotypes (Figure A1; Table S6). Results show that the term fatty acid metabolism was significantly observed in the highly embryogenic genotype (Figure A1b).

The significant role of fatty acid metabolism in embryogenic transdifferentiation process has been investigated. During cotton SE, genes related to fatty acid biosynthesis were upregulated in embryogenic callus compared to non-embryogenic callus. The results indicated that fatty acids accumulated during embryogenic callus development [15]. The proteomic analysis indicated that fatty acid biosynthesis and metabolism was one of the top pathways involved in cotton somatic embryogenesis, confirming the transcriptomic results on the important role of the fatty acid metabolism in cotton SE [19]. In addition, many fatty acid biosynthesis and metabolism related proteins, such as ‘cytochrome P450 86B1-like’ and ‘cytochrome P450 86A8-like’, were differentially accumulated during cotton SE. In the fatty acid biosynthesis pathway, these proteins were up regulated in PEC, implying the important contribution to the SE initiation in cotton [6]. The results above were consistent with our previous report that a lipid transfer protein (SELTP) drives the totipotency of somatic cells in cotton [43].

In tobacco cell cultures, the ectopic expression of *LEAFY COTYLEDON* (*LEC*) genes induced accumulation of fatty acid biosynthetic enzymes [64]. Most of genes acting in fatty acid and lipid biosynthesis were globally upregulated in *LEC* overexpression lines [65,66]. In the tree fern *Cyathea delgadii*, a comparative proteomic analysis was performed in stipe explants undergoing direct SE. The differentially regulated proteins during early SE were assigned to seven functional categories, including fatty acid metabolism [67]. The acetyl-CoA carboxylase (ACC) activity was crucial to SE process, which is a key enzyme in oil biosynthesis and catalyzes the first step in fatty acid biosynthesis [68]. A proteomic analysis also reported that energy production is increased during *Quercus suber* somatic embryogenesis probably to participate in the synthesis of primary metabolites such as fatty acids [69]. The amount of fatty acid may be used as a marker of embryogenic potential when promising cell lines of *Siberian larch* are screened in the stage of early embryogenesis [70]. The fatty acids, which affect cell function and growth patterns, appear to be a part of the thidiazuron action pattern and may play an important role in inducing regeneration [6]. In this study, some special genes were identified to be involved in fatty acid metabolism. Of these, *KCR1* (Very-long-chain 3-oxoacyl-CoA reductase 1) and *PAS2* (Very-long-chain-3-hydroxyacyl-CoA dehydratase 2) genes were significantly upregulated during embryogenic transdifferentiation in LM, *ECR* (Very-long-chain enoyl-CoA reductase) gene was significantly upregulated during embryogenic transdifferentiation in YZ (Table 2). Very long chain fatty acids are essential for many aspects of plant development, including cell proliferation. They were speculated to be required for polar auxin transport and tissue patterning during plant development [71]. Additionally, the *ADH6* (Alcohol dehydrogenase 6) gene was significantly downregulated during embryogenic transdifferentiation in YZ (Table 2). The expression of the *ADH* gene increased markedly in barley zygotic embryo development [72]. It was concluded that *ADH* gene expression in zygotic embryos was regulated by an ABA/gibberellin interaction. These conclusions demonstrated that fatty acid might frequently be associated with embryogenic transdifferentiation during cotton SE.

### 4.3. Pyruvate and Tryptophan Metabolism Involved in Embryogenic Transdifferentiation

Pyruvate is a derivative of pyruvate acid and plays a role in glycolysis, or sugar metabolism. Furthermore, tryptophan is an important precursor for auxin biosynthesis in plants. The structure of tryptophan is similar to that of IAA, which is common in higher plants. In the current study, KEGG enrichment analyzed the differences and dynamic changes of gene clustering in two cotton genotypes (Figure A1; Table S6). Results indicated that the pyruvate and tryptophan metabolism were also significantly observed in the highly embryogenic genotype (Figure A1b). 

The effects of pyruvate and tryptophan metabolism in SE process have been demonstrated. In cotton SE cultures, de novo transcriptome analysis investigated the genes expressed during SE and their expression dynamics using RNAs isolated from nonembryogenic callus and embryogenic callus [15]. Results showed that the differentially expressed genes related to pyruvate metabolites were identified between NEC and PEC [15]. Furthermore, iTRAQ approach was applied to study proteome changes and to identify differentially accumulated proteins between NEC and PEC. Through identification and annotation of differentially accumulated proteins, the key genes/proteins related to pyruvate metabolism involved in cotton SE were uncovered [19].

It has been shown that de novo auxin production via the tryptophan-dependent indole-3-pyruvate (IPA)-YUC auxin biosynthesis pathway was involved in cellular totipotency reprogramming during SE induction [73,74]. In sugarcane callus tissues, levels of different aliphatic compounds belonging to pyruvate metabolism changed significantly in response to different biochemical relationships between callus tissues and their nutrient media. Based on biochemical pathway analysis, it can be speculated that in PEC, alanine, valine, and leucine can act as possible precursors to carbohydrate synthesis through pyruvate metabolism [75]. Furthermore, several metabolites, including tryptophan present in embryogenic calluses [75]. In Maize cell lines, enrichment analysis of differential expressed genes revealed that pyruvate metabolism, biosynthesis of plant hormones, and fatty acid biosynthesis were identified to be three of the most affected pathways, indicating the conserved and important roles of pyruvate metabolism in the somatic embryogenesis process [76]. In the current study, some special genes were identified to be involved in tryptophan metabolism. Of these, *TAA1* (L-tryptophan-pyruvate aminotransferase) gene was significantly upregulated during embryogenic transdifferentiation in LM. *YUC4* (Probable indole-3-pyruvate monooxygenase) gene was significantly upregulated during embryogenic transdifferentiation in YZ (Table 2). During SE in *Coffea canephora*, there was an important increase in the expression of *YUC* and *TAA1* genes [77]. The *YUC* gene, an auxin biosynthesis enzyme, was highly accumulated in EC which leads to increase the level of free IAA [78]. This finding is supported by the evidence that biosynthesis of auxin through *YUCs* is involved in embryogenesis and induction of somatic embryos. The *YUC* gene might play an essential role in auxin signaling process to enhance SE. Moreover, *PPC* (Phosphoenolpyruvate carboxylase) gene, which was enriched in pyruvate metabolism was upregulated during embryogenic transdifferentiation in YZ (Table 2). Phosphoenolpyruvate carboxylase was found in increased abundance in zygotic embryos [79]. *PPC* gene can yield oxaloacetate that can be converted to aspartate, malate or other intermediates of the TCA cycle. Thus, this metabolic pathway maintains the pool of carbon residues necessary for the biosynthesis of oil and storage proteins that take place in later stage of embryo development. It can be speculated that *PPC* gene is involved in somatic embryogenesis through the TCA cycle. Results above reveled the significant role of pyruvate and tryptophan metabolism in embryogenic transdifferentiation during SE process.

### 4.4. Stress-Response Transcription Factors Involved in Embryogenic Transdifferentiation

Stress is an important signal regulator for cell dedifferentiation, which can induce the expression of a large number of transcription factors, cell fate reorganization and acquisition of embryogenic capacity [80,81,82,83]. In response to stress, somatic embryogenesis originated from embryogenic capability acquisition, cell dedifferentiation and changes of gene expression patterns [84,85]. 

Plant cells with different types specifically responded to stress factors, and different stress treatments had different effects on somatic cells [82]. Developmental variabilities induced by stress explained to some extent that culture conditions can affect somatic embryogenesis in tissue cultures. In this study, several transcription factors (TF), such as bHLH, MYB-related, B3, NAC and WRKY were dynamically changed during different developmental stages of somatic embryogenesis in the two genotypes (Figure 4). Previous studies have confirmed that these transcription factors played an important role in responding to various stresses. They were differentially expressed under stress conditions and acted as a link between stress and the developmental pathway [50]. During cotton SE, genes related to stress response were identified and specifically involved in cotton somatic embryogenesis [29,30,86]. In addition, TF genes were also investigated during *Arabidopsis thaliana* SE induced by in vitro culture. Transcripts related with phytohormones and stress responses were found to be most abundant [87]. 

In the current study, some specific transcription factors were identified to be involved in embryogenic transdifferentiation, such as *Wind, ESR, WUS, LEC* genes. As an AP2/ERF transcription factor, *Wind1* (Wound induced dedifferentiation 1) was proven to upregulate the expression of *ESR1* (Enhancer of shoot regeneration 1) gene that encodes another AP2/ERF transcription factor, promoting Arabidopsis shoot regeneration [88]. Another important gene that marks embryonic cells was the transcription factor gene *WUS*, which was involved in the promotion and/or maintenance of totipotent embryogenic stem cells. Overexpression of *LEC2* led to activation of *YUC2* and *YUC4* genes, encoding auxin biosynthetic enzymes, suggesting that *LEC2*-mediated somatic embryogenesis might be achieved by an endogenous upregulation of the auxin biosynthetic pathway [89]. 

In addition, the interaction between stress and auxin played an important regulatory role in chromatin recombination and embryogenic capacity acquisition. 2,4-D is not only a plant growth regulator, but also a stress inducer. About half of the stress-related transcription factors were involved in *Arabidopsis thaliana* SE process induced by 2,4-D [87]. The auxin and stress response were found to be involved in cotton somatic embryogenesis via transcriptome sequencing during callus dedifferentiation and transdifferentiation processes [30]. The stress response was activated and regulated auxin signaling during cotton somatic embryogenesis [86]. Moreover, abiotic stress factors can also affect auxin synthesis, transportation and stability in various ways during morphogenic response [80,83]. However, the mechanism of the interaction between stress and auxin for embryogenic capacity acquisition needs to be further investigated.

## Figures and Tables

**Table 1:** Significantly representative SE-related DEGs enriched in DNA conformation change process during embryogenic transdifferentiation.

Gene ID	Gene Name	Description	Log2 (Fold Change)	
			LM_PEC/ LM_NEC	YZ_PEC/ YZ_NEC
Gh_A11G2024	IGHMBP2	DNA-binding protein SMUBP-2	−1.86	—
Gh_D09G2407	SRS2	ATP-dependent DNA helicase SRS2-like protein	−1.65	—
Gh_D11G1733	RECQL3	ATP-dependent DNA helicase Q-like 3	−6.37	−4.37
Gh_A05G2891	SYN1	Sister chromatid cohesion 1 protein 1	−3.24	—
Gh_A07G0113	NAP1	Nucleosome assembly protein 1	−1.01	—
Gh_A09G0152	SMC4	Structural maintenance of chromosomes protein 4	−4.98	2.74
Gh_A08G2038	CENPV	Centromere protein V	−2.63	−1.10
Gh_A05G2192	TFG2	Transcription initiation factor IIF subunit beta	−1.43	—
Gh_A05G3742	CSP1	Cold shock protein 1	−6.26	—
Gh_A07G0517	PHRF1	PHD and RING finger domain-containing protein 1	−1.57	—
Gh_A12G1650	ASCC3	Activating signal cointegrator 1 complex subunit 3	−1.02	—
Gh_D07G2021	CDC45	Cell division control protein 45 homolog	−2.74	—
Gh_A01G1890	HIS4	Histone H4	5.07	3.56
Gh_A02G1361	HIS4	Histone H4	4.55	2.42
Gh_A03G0342	HIS4	Histone H4	5.36	3.23
Gh_A08G2531	HIS4	Histone H4	3.61	1.72
Gh_A08G2548	HIS4	Histone H4	4.92	2.95
Gh_A10G0449	HIS4	Histone H4	4.17	2.23
Gh_A02G0490	CAPH2	Condensin-2 complex subunit H2	3.17	2.29
Gh_A02G1767	GYRB	DNA gyrase subunit B, chloroplastic/mitochondrial	2.10	-
Gh_A03G0513	SMC2-1	Structural maintenance of chromosomes protein 2-1	4.01	2.17
Gh_A03G0582	NRP2	NAP1-related protein 2	1.99	1.55
Gh_A06G1021	ZRANB3	DNA annealing helicase and endonuclease	1.10	-
Gh_A03G0737	MCM2	DNA replication licensing factor	2.45	1.70
Gh_A09G0025	MCM2	DNA replication licensing factor	4.83	3.86
Gh_A05G1256	MCM3	DNA replication licensing factor	3.44	-
Gh_A03G1940	MCM6	DNA replication licensing factor	5.18	2.27
Gh_A04G1267	HMGIY2	HMG-Y-related protein A	1.69	-
Gh_A07G2178	HMGIY2	HMG-Y-related protein A	2.60	1.51
Gh_A05G2910	DNA2	DNA replication ATP-dependent helicase/nuclease	3.85	2.54
Gh_A07G0948	IDM1	Increased DNA methylation 1	6.01	4.99
Gh_A07G1821	SNA41	Cell division control protein 45 homolog	6.21	2.52
Gh_A09G0431	FKBP53	Peptidyl-prolyl cis-trans isomerase	1.87	-
Gh_A09G1577	ORTH2	E3 ubiquitin-protein ligase ORTHRUS 2	2.37	2.67

**Table 2:** Significantly representative SE-related DEGs enriched in fatty acid, tryptophan and pyruvate metabolism during embryogenic transdifferentiation.

Gene ID	Gene Name	Description	Pathway Annotation	Log2 (Fold Change)	
				LM_PEC/ LM_NEC	YZ_PEC/ YZ_NEC
Gh_D02G0833	ECHS1	Probable enoyl-CoA hydratase, mitochondrial	Fatty acid elongation	−1.65	−1.29
Gh_A12G1958	PPT3	Palmitoyl-protein thioesterase 3	Fatty acid elongation	-	2.69
Gh_A08G1244	PPT1	Palmitoyl-protein thioesterase 1	Fatty acid elongation	−7.51	−2.61
Gh_A01G0045	KCS9	3-ketoacyl-CoA synthase 9	Fatty acid elongation	4.63	4.02
Gh_A03G0701	KCS4	3-ketoacyl-CoA synthase 4	Fatty acid elongation	−1.94	−2.45
Gh_A03G1143	KCR1	Very-long-chain 3-oxoacyl-CoA reductase 1	Fatty acid elongation	5.43	4.23
Gh_A11G3137	PAS2	Very-long-chain (3R)-3-hydroxyacyl-CoA dehydratase 2	Fatty acid elongation	3.45	2.92
Gh_A05G3048	ECR	Very-long-chain enoyl-CoA reductase	Fatty acid elongation	-	1.16
Gh_A10G1742	GPSN2	Very-long-chain enoyl-CoA reductase	Fatty acid elongation	−4.89	−2.61
Gh_A01G1527	LACS2	Long chain acyl-CoA synthetase 2	Fatty acid metabolism	8.41	6.67
Gh_A01G0167	LACS7	Long chain acyl-CoA synthetase 7, peroxisomal	Fatty acid metabolism	−1.90	−1.50
Gh_A01G0141	ACX3	Acyl-coenzyme A oxidase 3, peroxisomal	Fatty acid metabolism	−2.01	−1.50
Gh_A02G1616	ALDH3	Aldehyde dehydrogenase family 3	Fatty acid metabolism	3.67	6.33
Gh_D08G2387	ADH6	Alcohol dehydrogenase 6	Fatty acid metabolism	-	−1.37
Gh_A03G1194	TAA1	L-tryptophan—pyruvate aminotransferase	Tryptophan metabolism	5.76	3.69
Gh_A05G0035	YUC4	Probable indole-3-pyruvate monooxygenase YUCCA4	Tryptophan metabolism	4.85	7.68
Gh_A08G0657	YUC10	Probable indole-3-pyruvate monooxygenase YUCCA10	Tryptophan metabolism	−4.92	−2.40
Gh_D09G0992	AAO2	Indole-3-acetaldehyde oxidase 2	Tryptophan metabolism	−2.03	−2.98
Gh_A02G1509	CYP	Cytochrome P450	Tryptophan metabolism	-	−4.14
Gh_A05G2791	UGT	UDP-glycosyltransferase	Tryptophan metabolism	-	4.15
Gh_D04G0292	ALDH3I1	Aldehyde dehydrogenase family 3 member I1	Tryptophan metabolism	1.25	2.10
Gh_A01G0390	PPC	Phosphoenolpyruvate carboxylase	Pyruvate metabolism	3.86	2.07
Gh_D08G2350	PCKA	Phosphoenolpyruvate carboxykinase (ATP)	Pyruvate metabolism	1.64	1.20
Gh_A10G1783	PYK	Pyruvate kinase	Pyruvate metabolism	−1.91	−1.40
Gh_A09G2065	DLD	Dihydrolipoamide dehydrogenase	Pyruvate metabolism	2.42	2.18
Gh_A05G2129	DLAT	Dihydrolipoamide acetyltransferase	Pyruvate metabolism	-	1.26
Gh_A05G3511	ACSS	Acetyl-CoA synthetase	Pyruvate metabolism	−2.07	−1.82
Gh_D11G3501	AAT1	Acetyl-CoA acetyltransferase, cytosolic 1	Pyruvate metabolism	-	−3.92

## Supplementary Materials

Supplementary materials can be found at https://www.mdpi.com/2073-4425/11/5/519/s1. Table S1. RNA-Seq output data through filtering and mapping, Table S2. The expression levels (FPKM) of all transcripts during early SE, Table S3. Differentially expressed genes (DEGs) during early SE in the two genotypes, Table S4. K-means clustering of differentially expressed genes (DEGs) during early SE in the two genotypes, Table S5. Gene Ontology (GO) analysis of DEGs during embryonic redifferentiation process in the two genotypes, Table S6. KEGG enrichment analysis of DEGs during embryonic redifferentiation process in the two genotypes, Table S7. Transcription factors (TFs) during early SE in the two genotypes.
